# Exploring the motivational states of English learning among Chinese EFL learners at tertiary-level: A perspective of Directed Motivational Currents

**DOI:** 10.3389/fpsyg.2022.1041258

**Published:** 2022-12-30

**Authors:** Xiang He, Dandan Zhou, Chang Wu

**Affiliations:** ^1^School of Foreign Languages, Jiangsu University of Science and Technology, Zhenjiang, Jiangsu, China; ^2^School of Foreign Studies, Nanjing University, Nanjing, Jiangsu, China

**Keywords:** EFL motivation, Directed Motivational Currents, English language learning, IELTS training course, Chinese tertiary-level EFL learners

## Abstract

Under the framework of Directed Motivational Currents (DMCs), the present study aimed to explore the motivational dynamics of Chinese tertiary-level EFL learners’ English learning and to identify the possible parameters that influence Chinese EFL learners’ DMC-typed motivational states in their English learning. Data were collected from 10 focal Chinese tertiary-level EFL learners through reflective journal, trajectory equivalent modeling and semi-structured interview over a two-semester-long IELTS training course. The collected data were examined with thematic analysis and the findings indicated that: (1) Chinese tertiary-level EFL learners experienced the clear DMC-typed motivational surges during their journey of English learning; (2) Chinese tertiary-level EFL learners’ DMCs states were affected by various contextual factors which could be examined under three major themes, namely important others, instructional elements, and the exam pressure. The findings not only add to the literature on the validity of DMCs construct in the Chinese EFL context, but offer implications about how to facilitate the DMC-typed motivations in the classroom language instruction.

## Introduction

Motivation has long been considered as a vital factor influencing learners’ attitudes toward multilingual and multicultural education (Ushioda, 2006; Moratinos-Johnston et al., 2019; Zheng et al., 2019). During the past half century, L2 motivation has been widely explored in the fields of educational psychology and SLA (Al-Hoorie, 2017; Al-Hoorie and MacIntyre, 2019) and various models of L2 motivation have been proposed (e.g., Williams and Burden, 1997; Dornyei and Otto, 1998; Dörnyei, 2005). The integrative and instrumental motivation model (Gardner, 1979, 1985, 2010) was the most popular theory at the earlier stage, and L2 motivation was explored by focusing on the social environment from a macro-perspective. At the second stage, some cognitive-situated and classroom-friendly models were put forward from a micro-perspective and some key motivation conceptions were proposed including the Self-determination theory (Deci and Ryan, 2008), self-efficacy (Bandura, 1997), and causal attribution (Weiner, 1985). The present stage was characterized by the proposal of Directed Motivational Currents (DMCs) to address the dynamics of motivation surges. Dörnyei et al. (2014) defined DMCs as a “prolonged process of engagement in a series of tasks which are rewarding primarily because they transport the individual toward a highly valued end” (Dörnyei et al., 2015a,b, p. 98). As a motivational phenomenon with distinguishing features of its intensity and sustainability, DMCs can serve as a powerful drive to support and energize L2 learners’ motivated behaviors in the long term, and assist L2 learners to be absorbed in a powerful motivational flow, bringing them closer to their idealized goals.

Up till now, a growing body of studies (e.g., Henry et al., 2015; Ibrahim, 2016a; Ibrahim, 2017; Muir, 2020; Safdari and Maftoon, 2017; Selcuk and Erten, 2017; Zarrinabadi and Tavakoli, 2017) have been conducted around DMCs, and the relative findings have provided confirmatory evidence for the significance of DMCs in L2 learning process. However, there still exist some gaps in the understanding of such a novel motivational construct. One possible gap concerns the little attention attached to the specifics of DMCs sustainability, and few studies to date have explored the factors contributing to the longevity of language learners’ motivational currents. Theoretically, DMCs can overcome the tendencies and temptations that may distract individuals from pursuing their vision-oriented goals, yet those caught up in DMCs are still prone to experience temporary motivational fluctuations. DMCs need to be re-triggered in the vicinity of distractions (Ibrahim, 2020; Muir and Gümüş, 2020) in order to channel the individual toward the future target successfully. In this regard, there is a need for the real-time analysis of the whole DMCs journey of EFL learners who are currently experiencing DMCs in educational settings. It is crucial to uncover the whole process of such a motivational phenomenon and the findings of such studies can provide language teachers insights about how to enable EFL learners to be caught up in DMCs experiences with suitable language instruction.

Motivated by the considerations above, the present study attempts to delve into the real-time process of EFL learners’ motivational experience and identify the factors contributing to longevity and permanence typical for DMCs experience.

## Literature review

### The major components of the DMCs

There are three core components of DMCs, namely goal/vision-orientedness, a salient and facilitative structure, together with positive emotionality (Dörnyei et al., 2014, 2015a,b, 2016). The three components need to be present and properly balanced so that learners’ highly-charged motivational pathways could be generated (Dörnyei et al., 2014).

Goal/vision-orientedness means a person’s directed motivational current “transports the individual toward a highly valued end” (Dörnyei et al., 2016, p. 98) without ending up at a random destination. Goals and visions can direct individuals’ efforts and energies toward doing specific tasks, resulting in the greater likelihood of goal attainment (Muir and Dörnyei, 2013; Henry et al., 2015; Dörnyei et al., 2016). Despite the generally indiscriminate use between goal and vision in most studies, the term of goal is more abstract with reference to the cognitive concept of the desired outcome whereas the term of vision is quite personalized, referring to the mental image associated with actual goal achievement (Dörnyei and Kubanyiova, 2014) or “the imagined reality of the goal experience” (Dörnyei and Chan, 2013, p. 454).

A salient and facilitative structure is characterized by three key features. To begin with, this facilitative structure provides a specific identifiable starting point for learners’ motivation to be stimulated or initiated from (Henry et al., 2015). Without an initial launch of triggering factors, no motivational surges would naturally happen (Dörnyei et al., 2016). As for the L2 learning, the ownership of given language tasks, together with students’ confidence and abilities to complete the task, is the necessary stimulus for their task engagement, serving as the initial triggering power of students’ motivational surges. The other features lie in the fact that this facilitative structure can boost the progression of the motivated behavior by creating a behavioral routine (Muir and Dörnyei, 2013; Dörnyei et al., 2014) and the regular progress checks (Dörnyei et al., 2015a,b, 2016). Behavioral routines refer to the common, routinized, effortless, or unreflective thoughts or behaviors when a person is involved in language learning and use, which are not fueled by volitional control or motivational processing (Muir and Dörnyei, 2013). By executing behavior routines across time, learners can enter a semi-automatic process and such effortless automation can ensure a given motivational momentum to be maintained. Progress checks serve as the conscious markers to evaluate learners’ performance, pinpointing the pathway toward success (Zarrinabadi et al., 2019). Sub-goals are generally formulated to check learners’ progress, creating a pathway for the motivational energies to go through.

Positive emotionality is an essential part of the affective dimension of DMCs, however, the positive emotionality resulting from DMCs is quite different from the intrinsic pleasant feelings concerning a hobby or other entertaining activities (Dörnyei et al., 2014, 2016). The positive feelings of a learner caught up in DMCs may derive from the happiness of approaching the goal by taking every step (Dörnyei et al., 2014, 2016) rather than completing the task. Consequently, such a positive emotional loading may generate the increasing energy and channel the motivational currents toward the target goal (Muir and Dörnyei, 2013). In language learning, “tasks and activities that might otherwise have been effortful, mundane and even tedious are approached with an effortless outflow of energy, and can generate deep-seated feelings of personal fulfillment” (Henry, 2020, p. 6), override perceived tiredness and boredom, keep a person’s motivational intensity (Henry et al., 2015) and produce learners’ positive emotionality as well.

### Research on DMCs in L2

Early empirical studies conducted mainly focused on validating the central components of DMCs by seeking evidence from adult learners with diverse language backgrounds. The first systematic empirical study was carried out to explore the motivational behavior changes in the Swedish-as-L2 learners (Henry et al., 2015). It was found that the Swedish migrants’ intensive motivational surges were characterized by the three core components of DMCs. Zarrinabadi and Tavakoli (2017) examined the active motivational behaviors of pre-service EFL teachers in Iran and figured out the three key components of DMCs among the Iranian EFL teacher participants. Likewise, Zarrinabadi et al. (2019) explored the highly motivated Iranian EFL learners and detected all the core constituents of DMCs in the participants’ motivational states. In the Chinese EFL context, Chang (2017) investigated 10 English postgraduates’ motivational dynamics and the identified properties of the participants’ motivational experiences were in line with the defining characteristics of DMCs. Following the same trend, Ning and Cai (2019) provided empirical support for the validity of DMCs construct with the interview-data from four focal Chinese participants. In a wider learning context, Muir (2020) conducted a large-scale online questionnaire study with the participants from 71 countries and found that a considerable part (39%) of participants reported the intensive motivational experiences with the distinctive features of DMCs.

The past 3 years witnessed a noticeable shift from the validation of the core components of DMCs construct to the exploration of the impacting factors that trigger the emergence of language learners’ DMCs experiences. For example, it is found that teachers’ comments, family members’ encouragements, and the willingness to talk with a foreigner were the potential external triggers, while some unhappy experiences in English learning like a failure to get understood was the possible internal stimulus (Zarrinabadi et al., 2019). The contextual factors impacting learners’ DMCs states included the language classroom atmosphere and the exam pressure (Sak, 2019), the contagion of others’ goals or ideas, as well as some social-situational factors (Zarrinabadi and Khodarahmi, 2021). Some psychological variables were also identified to affect the EFL learners’ DMCs experiences, like learners’ personality (Sak, 2021), self-efficacy and agency (Pietluch, 2018, 2021), personal best, buoyancy, and evaluation apprehension (Jahedizadeh et al., 2021),

As for the pedagogical applications of DMCs in language education, Henry et al. (2015) and Ibrahim (2020) discussed the theoretical importance of DMCs in directing language learners’ motivated behaviors, while other empirical studies examined whether language instruction could stimulate the real-time DMCs. Watkins (2016) made the first attempt to investigate the possibility of inducing 25 Japanese undergraduates’ motivational surges with a curriculum centered on the hallmark characteristics of DMCs, and the finding indicated that a carefully tailor-made language curriculum may promote leaners’ DMCs-like engagement. Based on the trajectory of three Chinese college EFL leaners’ self-concept development in spoken English, Fu (2019) proposed some DMCs-boosting recommendations for oral English instruction. Pietluch (2021) conducted a teaching experiment on 16 adult EFL learners and the outcome revealed that the language teaching curriculum with the efficacy-building characteristics may generate the prospective DMCs. At the group level, Ibrahim and Al-Hoorie (2018) found that the construction of group identity, the personally-related significance of the group project, along with the opportunity for leaners’ autonomy were the vital parameters to boost the motivational currents of group learners. Similarly, Garcia-Pinar (2022) conducted the intervention experiment on four undergraduates and found that the clearly-defined and personally meaningful tasks could generate the group’s motivational currents.

In accordance with the new surge of “dynamic turn in SLA” (Dörnyei et al., 2016, p. 1), more emphases have been recently placed on the dynamic and long-term aspect of DMCs. Some scholars began to explore the changing patterns of learners’ DMCs states from a dynamic perspective. For instance, Selcuk and Erten (2017) compared the patterns of motivational changes in two Turkish tertiary-level EFL learners caught in DMCs. In the Chinese EFL context, Chang and Zhang (2020) examined the changing patterns of listening motivational sates in five Chinese EFL listeners. The findings highlighted the importance of the pursuit of a visionary objective on the learners’ overall motivational performances. In a recent study, Chang (2021) carried out a multidimensional and longitudinal exploration to analyze the complex properties of Chinese tertiary EFL learners’ DMCs experience in their English language learning.

Previous empirical studies have greatly expanded our knowledge of DMCs construct, yet there is still room for the exploration of the whole journey of DMCs states. Much remains to be known about how DMCs experience could be promoted or inhibited by the context-related and situation-specific parameters in L2 learning environment. Moreover, although “time” is the primary focus of DMCs, most previous studies have retrospectively explored the DMCs experiences occurring in different contexts in the past (Ibrahim, 2016b). The retrospective accounts, however, may simply represent “the *post hoc* rationalization by participants as to how it would make sense for motivation to develop over time” (Jahedizadeh and Al-Hoorie, 2021, p. 533). It is crucial to investigate the immediate process of the DMCs journey in order to explore the real DMCs state (Al-Hoorie and Al Shlowiy, 2020; Vitta and Al-Hoorie, 2020). In this regard, there is a need for the real-time analysis to uncover the whole process of DMCs experiences.

Given that DMCs are generally initiated by something consciously specific and DMCs can be directly linked to the project-based language teaching (Dörnyei et al., 2016), the current study is designed to investigate the possible parameters leading to the emergence of DMCs in Chinese tertiary-level EFL learners while attending the IELTS training-based English learning. To be more specific, this study aims to address the following two research questions:

RQ1:*To what extent do the fundamental components of the DMCs construct account for Chinese tertiary-level EFL learners’ intensive motivational currents?*

RQ2:*What are the factors contributing to the permanence of Chinese tertiary-level EFL learners’ motivational currents in their English learning?*

## Methodology

### Research context

This study was conducted in a provincially comprehensive University located in Jiangsu province of mainland China. This university has the Sino-Australian cooperative project for the major of business administration in the school of economics and management, where students pursuing a bachelor’s degree of business administration will spend the first 2 years studying in the mainland of China and the latter 2 years in Australia. To pass the IELTS test with the minimum average score of 6 in listening, speaking, reading, and writing is compulsory for all students in this major before getting their oversea study in Australia. Accordingly, this university provides a special training course to help the freshmen prepare for the IELTS test. The IELTS training courses last two semesters in the first academic year, containing four sections of listening, speaking, reading, and writing. Four teaching periods (one teaching period has 45 min) are set aside, respectively, for listening, speaking, reading, and writing, respectively, each week.

The IELTS training courses are normally conducted through the traditional face-to-face classroom instruction. Due to the breakout of COVID-19 epidemic at the very beginning of 2020, this training courses were forced to be conducted *via* online teaching for epidemic prevention and control in the second spring semester of 2020. Various synchronous and asynchronous online teaching platforms were utilized to carry out the necessary teaching practices. In this study, IELTS training courses were carried out in the traditional classroom setting in the first autumn semester while the second part of IELTS training coursed were conducted under the online environment.

### Research design

This study adopted a qualitative research method design, including students’ reflective journal, students’ self-assessment of English learning motivation, and the semi-structured interview. Prior to the investigation, ethical clearance was obtained from the ethics commission of Jiangsu University of Science and Technology for the approval of undertaking the whole procedures of the study. To achieve a holistic view about the DMC’s trajectory, including its launch, progress, and possible turning points, the participants were required to make the self-assessment of their English learning motivations by scoring within the range of 0 ~ 100 points biweekly. Then, all the self-ranking scores were plotted into a graph line within the same coordinate axis. At the same time, the participants made the reflective journals to account for the drive of their motivation currents at fortnightly intervals. Thereafter, semi-structured interviews were conducted and thematic analysis was implemented in order to figure out the possible influencing factors of the participants’ DMC-typed motivation changes.

### Participants

Considering that DMCs are special motivation currents with distinguishing features, not all motivation experiences could be treated as DMCs. To select the participants who were most likely to have the DMCs experience and track down their real motivation flows, a systematic procedure utilized in previous studies (Henry et al., 2015; Zarrinabadi and Tavakoli, 2017; Zarrinabadi et al., 2019) was adopted. The participants were recruited with three stages as follows:

To begin with, the first author, also one of the key instructors of the IELTS training courses, explained the research purpose to the students at the very beginning of the course after getting the approval from the university administration. A total of 98 students majoring in business administration set their English learning orientation with the expected IELTS scores. With the combination of students’ English learning target, their classroom activities plus their assignment performances during the first 2 weeks, 30 students were initially selected and invited for a preliminary interview. After clearly informed about the voluntary participation, as well as the anonymous and confidential responses, the chosen students signed the informed consent and agreed to participate the research. All the 30 students were required to make the self-assessment by scoring their English learning motivation and take down the reflective journals to account for the possible changes of their motivation in details as much as possible every 2 weeks.

At the second stage, the researchers plotted the 30 students’ self-rating scores into graph lines, respectively, within the same coordinate axis and examined the participants’ motivation graphs to identify unusually intense motivation surges. After comparing the students’ motivation lines, the researcher selected 10 students whose motivation graphs resembled the core features of DMCs and invited them for the next round of interviews. At the third stage, these 10 students were selected as the final participants. There were six males and four females, with mean age of 19.7 (SD = 0.67). The participants submitted their reflective journals and were interviewed about their motivation experiences based on the key tenets of DMCs.

### Instrumentation and procedure

Apart from the normal practice integrated between graphic elicitations and the interviews adopted in previous DMCs research (e.g., Henry et al., 2015), reflective journal was utilized to collect data. The graphic lines helped the participants to focus on the interview topic so that they could recall their motivational experiences and generate more deep thoughts. The reflective journal could help the researcher dig out more internal and external factors to impact the participants’ DMCs, resulting in more comprehensive data than what interviews alone would have generated. In a word, the research data were collected through reflective journal, motivational graphs, and semi-structured interview.

#### Reflective journal

The participants wrote at least one reflective journal in details to account for the changes of English learning motivation every 2 weeks and they handed in the reflective journals to the researcher *via* E-mail. The participants wrote the reflective journal between the sixth week and the nineteenth week of the first semester, and continue throughout the second semester from week 1 to week 17 (winter vocation was excluded). Ten participants submitted 140 reflective journals with a total of 9,985 words at the end of the second semester.

#### Motivational graphs

Given that “Retrieval of a past experience involves a process of pattern completion… in which the remembered pieces together some subset of distributed features that comprise a particular past experience, including perceptual and conceptual/interpretive elements” (Schacter and Addis, 2007, p. 774), participant’s retrospectively self-plotted graph over the past time would most likely fail to capture some subtle fluctuations (Ibrahim, 2016b). To overcome the potential weakness, the present study tried to create the real-time motivational graph over the participants’ English learning journey. The participants were required to make the self-rating of their motivation experience while taking down the reflective journals. Then, the researchers plotted the participants’ self-scores into motivational line graph as a prompt for the subsequent interview. It is hoped that such real-time analyzing approach may shed more lights on the whole process of DMCs development over time (Jahedizadeh and Al-Hoorie, 2021).

#### Semi-structured interviews

The semi-structured interviews were conducted by the first author, who had the prior experience in DMCs research. She was the leading instructor of IELTS training courses and had a quite harmonious relation with the individual participants, which could ensure the success of the interviews. The semi-structured interview was conducted *via* WeChat video-calling, through which the Quasi face-to-face communication could be achieved between the researcher and the participants. The interviews were guided by a list of open-ended questions, appropriated worded and targeting at the participants’ motivational experience regarding English language learning and possible influencing factors. Before implementing the formal interview, a pilot interview was first carried out on two different individual participants to verify the interview questions. The revised guideline encompassed the questions of the DMCs construct relating to participants’ English learning performances, together with the factors to launch those motivational changes (see Appendix 1). The interviews were conducted in the semi-structured way, namely the early arranged questions could be chosen at random or additional questions could be asked if it was necessary or seemed interesting (Dörnyei, 2007).

Throughout the interviews, participants’ motivational graphs were used as the stimuli to help them recall and to account for their motivational changes in English learning, thus the turning points in the graphs were particularly significant (Beijaard et al., 1999). To avoid the misunderstanding resulting from language barriers, the interviews were conducted in Chinese and were recorded with the informed consent of the participants. The individual interview lasted from 20 to 30 min, adding up to 4 h in total. The participants’ audio recordings were transcribed through ifyrec^1^ into texts. The transcribed texts were double checked based on the recordings and any inappropriate transcriptions were modified. Altogether 40,500 words were coded in the final transcribed text.

The whole procedure of the research was carried out as follows: see Table 1.

**Table 1:** Procedure of research design.

Procedure	Time	Participants’ tasks
Stage 1	Week 6 (first semester)*	To be informed of the research purpose and the specific procedure and fill in the consent form.
Stage 2	Week 6–19 (first semester) Week 1–17 (second semester)	To make the self-assessment of the English learning motivation and take down at least one reflective journal every 2 weeks
Stage 3	Week 14 (second semester)	To submit the reflective journals and attend the interview

*The freshmen registered at the university 3 weeks later than the seniors. They got the military training for 2 weeks before attending the academic lectures, thus the new academic semester began in the sixth week.

### Data analysis

The participants’ reflective journals and interview transcripts were analyzed by adopting approach of Braun and Clarke (2006) for thematic analysis. Given that DMCs construct is the theoretical framework and that the research purpose is to seek out the core features of DMCs as well as the possible influencing factors contributing to the emergence and permanence of DMCs, both the deductive thematic analysis and inductive thematic analysis were conducted to code and analyze the collected data through the lens of the DMCs construct. The data analyses were implemented through the following four steps.

To begin with, two researchers familiarized themselves with the contents of the data by reading the transcript independently. They conducted the initial coding of the data through searching, reviewing, defining, and labeling the areas related to the key features of DMCs. They highlighted the emerged themes with different colors, to be more precise, red for goal-orientedness, pink for positive emotionality, green for DMC structure, and blue for possible influencing factors.

In the second step, the researchers re-read the text data and made a second coding of the transcripts, concentrating on the phrases or structures signifying the factors to trigger the participants’ DMCs and highlighting them in yellow. Then, the researchers grouped the coded theme according to three core components of DMCs and the impacting factors. The researchers also made some notes in the margins of the transcripts with the coding terms/phrases. In the following step, the two researchers made a discussion to settle the disagreement in the definition and label of the coded themes.

In the fourth step, the credibility and dependability of the data analysis were checked with three evaluative criteria, namely member-checking, intercoder reliability checking, and external audit (Denzin and Lincoln, 2011; Creswell, 2012). To perform the member-checking, the first author contacted the participants personally *via* Wecat and read the coded data to the participants to ensure that the participants’ thoughts have been accurately interpreted. The author made some modifications of those over-interpretations or biased reports of the participants’ experience. To check the intercoder reliability, a teacher with the previous experience of DMCs study and qualitative research was invited to code some parts of data. Formula of Miles and Huberman (1994) was adopted to calculate the inter-rater agreement, resulting in the acceptable level of the inter-coder agreement (92%). In the end, the external audit was conducted by the same teacher. After looking through the final report, he made some recommendations about the interpretation of the coded data as well as the writing illustration.

## Results

In this section, the participants’ motivational graphs are presented first, followed by the results about the key features of the DMCs of participants’ motivational experiences. In the end, the findings of the influencing factors contributing to longevity and permanence of the DMCs are put forward.

### Participants’ motivation trajectories

**Figure 1:**
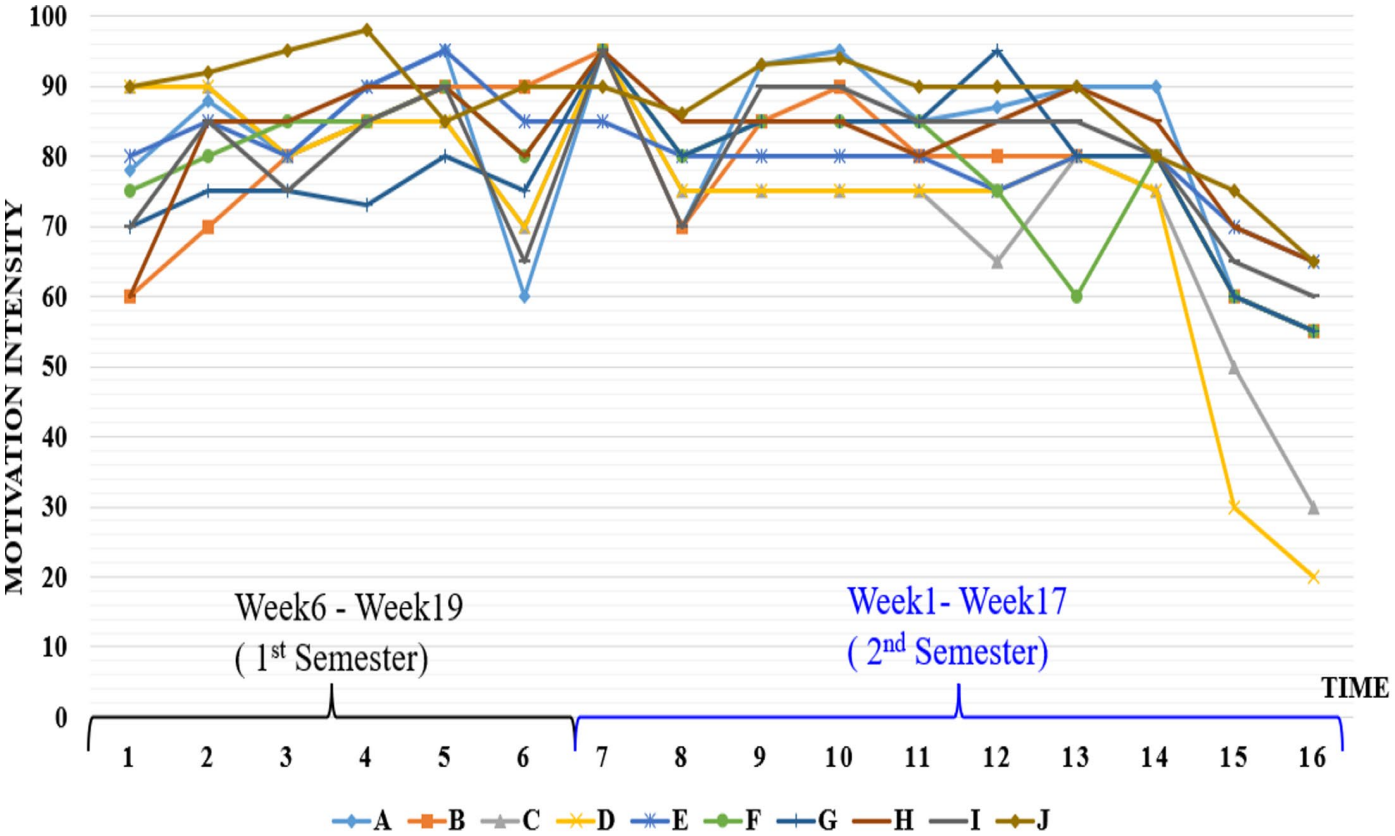
Trajectories of 10 participants’ motivation journey.

In Figure 1, the ordinate axis refers the self-assessment of the motivation intensity scored from 0 to 100 while the abscissa axis indicates the experiment time. The data were collected from the 6th to 19th week of the first semester to the 1st–17th week of the second semester (winter vocation was excluded), lasting 32 weeks altogether. The participants made the bi-weekly self-scoring of their motivation experiences and wrote the reflective journal every 2 weeks, and the interval of 2 weeks was set as the basic time unit in abscissa axis.

It can be clearly seen that the motivational graphic lines, despite some fluctuations, are characterized by the flows with the high intensity akin to the strong currents in the ocean channeled toward the final destinations during the majority part of the whole process. Participants’ motivation trajectories indicate that they had experienced the highly intensive and enduring motivational surges in pursuit of their special goals while attending the IELTS training courses. Consequently, it is safe to conclude that individual participants experienced the powerful goal-oriented motivational surges, namely Directed Motivational Currents (DMCs). Empowered by the strong DMCs, the participants had been constantly injected with strong motivational drives and thus they were energized and propelled in the direction of their specific targets on their English journey.

### Key features of participants’ DMCs

Under the theoretic framework of DMCs construct, the deductive thematic analyses were conducted to seek for the themes representing the key aspects of DMCs in participants’ reflective journal and interview transcripts. Three core themes emerged in analyzing the data of participants’ English motivational experiences (see Appendix 2) and the coding scheme was listed as Table 2.

**Table 2:** The coding scheme for the core features of DMCs in participants’ motivation stories.

Data extract	Coded for	Theme
…to speak English fluently …to be good at English writing …to improve English writing skills …to sing beautiful English songs …to talk with foreigners confidently … to find a well-paid job in a foreign company… …attending a western university	To improve English proficiency To seek a good job To get a further education	Goal/Vision
…repeated these activities every day. …practice as regularly as I can. …reading aloud and imitating… every day. … got up early and practiced reading English every morning. … teacher often gave me positive feedback… …teacher’s comments…push me forward. …better scores were the convincing signs for my progress…	The recurring behavioral routines The regular progress check	A salient facilitative structure
…felt happy and excited… …pushing forward felt good… …felt great to make progress …was confident… …looked energetic and passionate… …enjoyed moving forward… …felt fulfilled and pleasant… …had a meaningful life… …full of positive feelings… …looked joyful… …the best time…	Satisfaction and enjoyment in making progress	Positive Emotionality

As pointed out by Braun and Clarke (2006), the rich description of the data is important for the thematic analysis in order to provide the detailed and nuanced account of the theme. The following part will present the in-depth analysis centered around the above three core themes within the data set of the participants’ English learning experiences.

#### Goal/vision-directedness

As a clearly-defined superordinate goal, target or outcome coupled with a vision of its accomplishment, goal/vision-directedness can provide individuals with the orientation for the learning process and channel the daily behavior toward the goal attainment (Henry et al., 2015).

All participants expressed their clear ultimate goals for English learning. For most participants, the desire to improve English proficiency and to pass the IELTS test was their major motives to learn English. For instance, participant B said: “I am eager to pass the IELTS test. I want to speak fluently, listen well, read well, and be good at writing. I must work very hard.” When talking about his desire to improve the speaking ability, Participant E said: “Being able to speak English as fluently as a native speaker is my big dream.” Besides the oral communicative competence, participant F also expressed his hunger to enhance the English writing ability:

*English writing is always a big headache for me. There are two writing tasks in the IELTS test. That’s too bad. I want to improve my writing skills. I hope I can make progress in writing after attending the IELTS training.*

As for the long-term vision, participant A mentioned his ideal future job.

*I wanted to find a well-paid job in a famous foreign company. I thought IELTS training could help me to pass the IELTS test and give me the chance for further study abroad. I hope the oversea training and education background could help me to get an ideal job in the future.*

Besides the future job, some participants’ learning targets were associated with their self-images of being qualified English speakers. Participant G, for instance, talked about her vision to sing English songs:

*I love English songs and English movies. I think singing English songs is quite cool. I wish I could sing beautiful English songs one day. While attending the listening and speaking courses, I tried very hard to catch and imitate the native speakers’ pronunciations and intonations.*

Similarly, some participants envisioned themselves to be successful English users, and enjoyed the ideal future-self by considering it as one part of their future core identities. Both participant C and participant I shared their thoughts about the magic driving power of realizing their imagined vision:

*Whenever I encountered the obstacles, I would close my eyes to imagine myself to be in a foreign university, discussing with my classmates or giving the academic speech at the international conferences. This special daydream had the magic power to drive me to move on.* (Participant C)

*Our English teacher once invited several outstanding alumni to share their oversea learning experiences with us. They were so charming and excellent, and their stories encouraged me a lot. I wanted to become their kind of person someday! This beautiful idea could cheer me up and push me forward whenever I felt down.* (Participant I)

Based on the above analysis, it can be concluded that all participants had a clearly-defined goal in English language learning. Someone may fight for improving a particular English skill or the overall English proficiency while others may struggle for an ideal job or a wonderful future identity. Such findings were in line with the “permanent presence of a clearly-defined goal” of the DMCs construct (Henry et al., 2015: 331). These well-defined goals set the orientation for the DMCs and then propel the participants to move toward their imagined future-self by connecting the clear end goal with a vision of its accomplishment. At the same time, the goal is enriched by the imagined reality of the actual goal achievement (Dörnyei and Kubanyiova, 2014). For a language learner in DMCs, his future-oriented image of being successful in target language becomes part of his core identity (Dörnyei and Kubanyiova, 2014) and gets connected to practical living experiences (Ushioda, 2011) as well as to his “transportable identity” (Zimmerman, 1998). Under the guidance of the future-oriented self-image, the participants caught in DMCs can be propelled from the real setting into an imaginary situation where they can get engaged in social interactions with people from different nationalities, whether in the form of the dreamed occupation for participant A or the imagined bilingual identity for participant C.

#### A salient facilitative structure

The facilitative structure of DMCs can lay out the path and set aside the stages for the accomplishment of the end goal (Henry et al., 2015). There are three defining components of the facilitative structure of DMCs, including the recurring behavioral routines, the regular progress checks, and the discernible starting points and ending points. Such distinctive features of DMCs structure clearly emerged when analyzing the participants’ motivational stories.

The recurring behavioral routines were identified as the individuals’ common and routinized behaviors in their language learning. There were fixed arrangements for different language skills in the IELTS training course. All participants reported that they had scheduled their timetable according to different training sections, ending up with their routinized learning habits. For example, Participant B described her daily activities at school in the first semester:

*I had a very tight schedule every day. I usually attended the lectures on different language skills in the day time and spent much time on vocabulary learning, reading, or writing on my own in the evening. I also listened to some English news broadcast before going to bed. I followed these routines almost every day, but I felt full of energy.*

In a similar way, Participant E mentioned his reading practice in the morning:

*I wanted to improve my listening and speaking ability. I went up early in the morning, reading English and listening to English news broadcast. It’s not easy but I managed to keep it as a habit.*

Apart from the big chunk of time, Participant G also talked out his arrangement of spare time:

*I shifted my cellphone’s language mode from Chinese into English. I also installed some English news apps on my mobile phone. Whenever I waited in line for meals or buses, I would enter the news website to browse the latest news. I benefited a lot from the authentic wording of various news reports.*

Due to the breakout of COVID-19 pandemic, the participants attended the online courses in the second semester. Compared with the campus environment, the online learning atmosphere at home was more relaxing. However, the participants caught in the English learning DMCs followed the timetable in the same way as they were on campus. Their intense motivations for English learning were so self-evident and noticeable even to the people around. Participant F noted his parents’ response to his learning attitude at home:

*My parents were quite surprised by my diligence. Every morning, I got up early and practiced reading English or listening to some English newscast rather than lying in bed late. My parents were very satisfied with my performance. They told me that all my hard work would be paid off and they were proud of me.*

Participant I also mentioned:

*My friends felt it quite unbelievable that I could concentrate on English study yet declined to play online games with them. When they finally realized that I was not kidding, they gave up inviting me for joining in the online games any more.*

All the participants’ stories seemed to indicate that the motivational currents can orient the individuals’ effort to be directly channeled to the goal achievement while other unrelated factors would become secondary (Henry et al., 2015). As a result, the participants caught in DMCs could repeat their similar behaviors without many conscious efforts, nor the need of being fueled by any volitional control (Muir and Dörnyei, 2013).

Apart from the recurring behavioral routines, regular progress checks in the language learning process can also track down learners’ sub-goals, orient the pathway for learners’ motivational energy, and push it to pass through toward the end goal. The progress checks of the DMCs construct can be achieved in different forms. In the present study, some participants got the progress checks from other people’s comments. Participant C, for example, explained how her teachers’ feedback stimulated her continued efforts:

*My oral English teacher often praised my growth in pronunciation and intonation. He told me that I was getting progress in oral English and I could get a satisfying score in IELTS speaking test if I went on my effort. That was really encouraging.*

Similarly, Participant D shared his happiness in getting teacher’s positive comments on writing:

*Our writing teacher had the habit of underlining the good parts of the writing composition in red and adding some comments, such as ‘Good point, I like it!’, ‘Very special perspective!’ or ‘Excellent!’. I was so excited to find these nice comments in my writing paper. My teacher’s positive comments convinced me that all my efforts counted and pushed me forward.*

Besides teacher’s written comments, Participant J also talked about his scores generated from the online automatic writing grading system^2^:

*The increasingly higher scores graded by Pigai system encouraged me a lot. These scores were just like the granting signs for my progress and stimulated me to get devoted to writing.*

For the language learners in DMCs, regular progress checks can be derived not only from significant others, but also from their internal perceptions as well. In this study, some participants’ perceived thoughts could be considered as the important markers for their progress. Participant H mentioned her self-perceived improvement as an impetus for her perseverance in practicing listening and reading every morning:

*I felt very delighted when I could gradually catch the flow of the native speaker. More importantly, I could be more confident to talk with my foreign teacher in English. I began to realize that I was making progress and that kind of feeling was so great. I was determined to go on my reading and listening practice in the morning.*

In a word, both the internally perceived progress and the external progressive feedback injected the participants with continued energy and power. Once the individuals’ self-confidence and self-efficacy get stimulated, the motivational impetus will orient and support their subsequent efforts toward the final goal (Henry et al., 2015).

The starting and ending points of most participants’ DMCs trajectories could be clearly identified from the motivational graphic lines in Figure 1. Most participants’ initial motivational currents were characterized by strong and sudden launches, while the ending parts were found to be abruptly dissipating for some participants and gradually ebbing away for other participants. With regard to the starting points, DMCs have clearly recognizable releases of motivational energy launches and the triggering factors vary from person to person and differ in nature. For example, participant C explained his desire to get oversea study *via* passing IELTS test was stimulated by his unexpected failure in the Chinese college entrance examination, whereas participant J mentioned how her drive to learn English well was triggered by her sister’s tough job-hunting experience due to her sister’s deficiency in English.

Once setting out from the starting point, DMCs may exist for certain periods of time and finally arrive at the ending points, with either a sudden dissipation of motivation current or a gradual fading away of motivation energy. Any individuals caught up in DMCs can have an acute awareness of such motivational decays. The participants in the study also reported their acute sense of motivational changes. Participant B, for example, confessed the decrease of her motivation when she thought she had accomplished her goals:

*I had passed the IELTS test and got my satisfying score. It cost me so much time and efforts to prepare for the IELTS test during the past year. I thought it was the time to put my efforts into other courses as well.*

Participant G expressed his frustration when he found that he may not go abroad for further study temporarily:

*Studying abroad was always my dream. But the wide spread of the COVID 19 pandemic forced me to make different arrangements for my further study. I was at a loss and felt rather demotivated.*

#### Positive emotionality

For the individuals caught in DMCs, positive emotional experiences may come from various sources, like strong personal pleasures, satisfactions, achievements, and performing the activities toward their identified ultimate goals (Dörnyei et al., 2016). In this study, participants’ positive emotionality may come from the fulfillment of some subgoals at different stages of the IELTS preparing journey. Participant J described how her confidence was developed with the realization of her subgoals:

*The whole journey was wonderful. To pass the IELTS test, I set several small targets for my English learning. I worked hard every day and tried to accomplish my sub-goals one by one. I was so happy to have my goals achieved gradually and the feeling of coming closer to my final dream was so exciting.*

The satisfying experience of making progress and achievements can make a positive impact on individuals’ motivations (Murphy, 2011), generating the subjective feelings of rightness in their activities and the enhanced strength of their competence. As participant B commented:

*I felt so great to make progress every day. From the initial fear of the IELTS test to my present confidence, I was pretty sure that I was doing the right thing. All my hard work has been paid off and I was determined to continue my efforts.*

For the individuals in DMCs, positive emotionality may also derive from the accomplishment of oriented-goals closely connected to their future identities and the actualization of personal potentials (Henry et al., 2015). In this study, some participants attributed their intense pleasure to the development of their potentials in pursuing future targets. Participant F, for instance, expressed his excitement in progress:

*English has always been my headache in senior high school days. I was so excited when I found I could memorize more difficult English words, speak longer English sentences, and have a better understanding of English lecture. I could feel my progress and now I believed that I could learn English well.*

In this study, the IELTS training courses were conducted through the online platform in the second semester. The lack of face-to-face communication and the spatial separation between instructor and learners may generate the monotony in language teaching instruction online. However, the potential of achieving envisaged goals and the attainment of sub-goals can provide the individuals with continue power to achieve positive emotionality (Dörnyei et al., 2016; Al-Hoorie, 2017), and assist them to combat possible tiredness and boredom in order to keep their motivational intensity (Henry et al., 2015). Participant G described his highly-motivated online learning behaviors as follows:

*Some students complained about the boredom and low efficiency of online learning. But I couldn’t agree with them. I didn’t have the time to get bored because I was always busy in completing various assignments. It was such a great experience to fight for my dream and I felt I had a very meaningful life.*

All participants’ comments about their motivational experiences indicate that their enduring activities in English learning were accompanied by the deep perception of positive feelings and emotions, including pleasure, enjoyment as well as satisfaction, which finally propel them to allocate more time and efforts to their goal attainment in English learning.

### Contextual factors affecting participants’ DMCs

**Figure 2:**
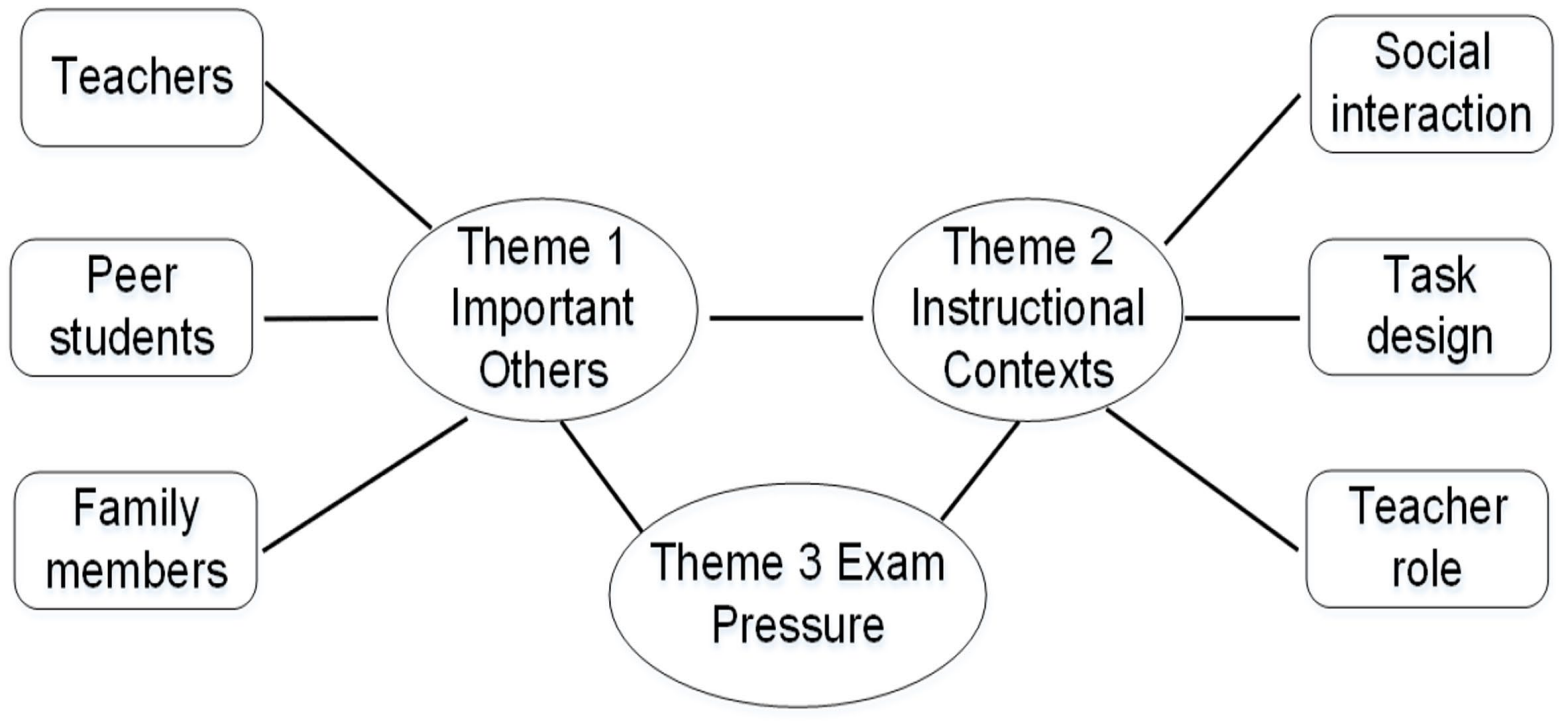
Thematic map of the influencing factors.

As illustrated in Figure 1, all participants experienced the clearly high motivation surges, yet not all of them began to be caught up in DMCs at the very start. It is also clear to find that all participants experienced some motivational fluctuations, indicating that however strong or specific future visions the participants had, their motivational currents were still integrated with some coexisting factors in their English learning contexts. The inductive thematic analyses were conducted to code the participants’ interview data, and three major themes were identified as the possible influencing factors on the participants’ DMCs experiences. The thematic map is illustrated in Figure 2. Considering the importance and necessity of supporting themes with detailed data extracts (Braun and Clarke, 2006), the following part will provide the in-depth analyses of three themes with relative interview excerpts.

#### Theme 1: Important others

Important others emerged from the data analyses as a noticeable theme. Language teachers have always been considered as one of the most important sources for language learners’ motivation (Hennebry-Leung and Xiao, 2020). Apart from teachers, family members, and friends have also been identified as the salient others to elicit learners’ DMCs (Zarrinabadi et al., 2019). In this study, the analyses of participants’ accounts yielded similar results, namely teachers, peer students, and family members were found to be the important others to trigger participants’ motivations to learn English well. Four participants expressed their gratitude for English teachers’ constant advice and assistance, which highly stimulated their motivational surges in English learning. Participant B made such comments:

*My oral English had a very beautiful British pronunciation. I wanted to be able to speak English as fluently as he did. My teacher helped me a lot by sharing the secrets of correct intonation and pronunciation. He even made some specific plans for my speaking practice. I was constantly pushed forward to speak well. I really appreciated my teacher’ help.*

Some participants also talked about the pushing power their peer students’ diligent performance has brought about. For example, participant D described his feelings in observing his roommate’s hard work in English study:

*My roommate, Jack, impressed me a lot. I was hugely attracted by his good learning habit and strong will to learn English. We shared the same dream of studying abroad. Whenever I became lazy, his full engagement in English learning always encouraged me and moved me forward.*

Participant F shared his experience about how he was intensely motivated by his elder sister while attending the lectures on online at home in the second semester. He made the comments as follows:

*Due to the epidemic prevention and control, both my sister and I stayed at home for online learning. My sister was a role model for me. She had such a passion and great perseverance in English learning. Her hard work and strong will encouraged me to try my best to learn English well.*

The above analyses reveal that important others around can trigger the participants’ DMCs in language learning, either in the form of positive comments, or activating the future goals in participants’ mind, lending support to the findings of previous study (e.g., Aarts and Dijksterhuis, 2003).

#### Theme 2: Instructional contexts

**Figure 3:**
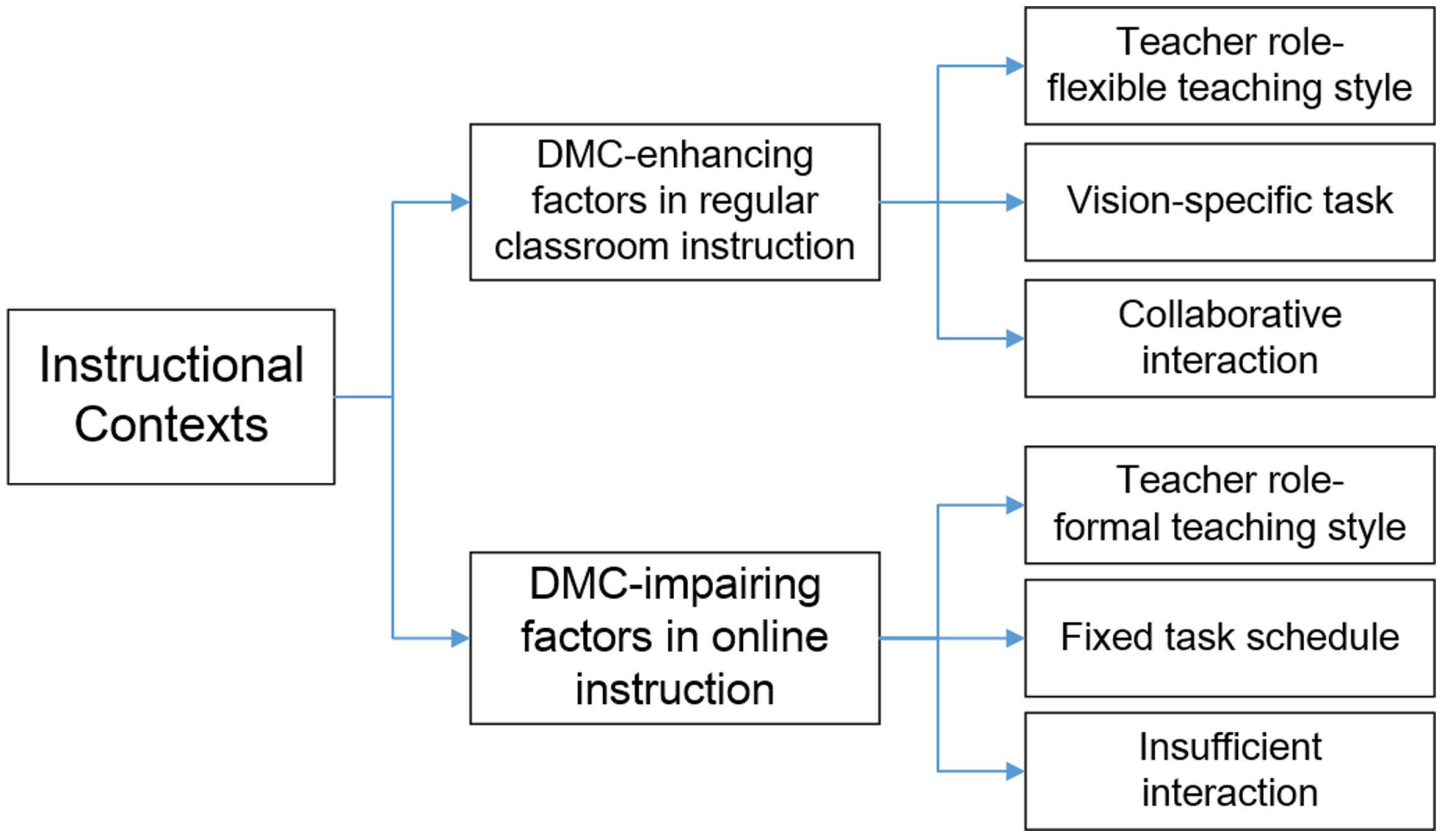
Directed Motivational Current (DMC)-influencing factors under different instructional contexts.

In this study, all participants attended the face-to-face classroom lectures in the first semester and the online lectures in the second semester due to the prevention and control of COVID-19. The results of the inductive thematic analysis revealed instructional contexts as the second salient theme to impact the participants’ DMC-related motivational experiences. Meanwhile, the micro-aspects of instructional settings were identified as the important subthemes, including teacher role, task design, and the social interaction (see Figure 3).

##### Teacher role

Teacher has always been considered to play a pivotal role in stimulating students’ motivation in regular classroom instruction, whereas such a motivation-eliciting role was found to diminish under the online learning environment in this study. Concerning teacher role, all participants expressed their preference for the flexible teaching style in regular classroom instruction compared with the tightly fixed teaching style in online setting. As participant G reported:

*In the traditional classroom, our teacher can conduct a variety of practices, especially in the English-speaking section. He may occasionally deviate from the normal procedure based on students’ speaking performance and offer immediate feedbacks either through his voice, facial expressions, or body gestures. In online setting, however, all the activities and teaching styles were pre-determined. I think it was ok to conduct English reading and writing instructions online, yet great barriers did exist for free communications online.*

In the light of participants’ views about the teaching styles under different instructional contexts, it seems reasonable to make the judgment that teachers’ greater flexibility in teaching styles and contents could smooth the course flow and enhance teaching efficiency, causing the promotion of students’ DMCs development.

##### Task design

As for the language tasks, most participants voiced their favors in the vision-specific tasks under the face-to-face instructional setting, as well as the inconvenience of striking speaking activities online, which indicates the significance of visibility in the English-speaking activity. Participant I compared her feelings toward the speaking activities under the two instructional contexts:

*I preferred the direct eye contact and immediate feedback from my teacher in the traditional classroom. Talking to my teachers and classmates on the computer screen was a very strange experience. I felt disconnected and couldn’t find a sense of belonging and engagement. I thought The speaking instruction online impaired greatly my English-speaking motivation.*

Such a finding seems to provide reasonable evidence to argue that learners’ motivation currents for certain target can gain momentum only after being nourished by relevant vision-specific activities.

##### Social interaction

When it comes to the social interaction, all participants reported their fondness of collaborative learning activities and the data analyses revealed that cooperative interaction was a major factor to work on the participants’ motivation changes within the instructional surroundings. Participant H shared her motivational experience in both the regular classroom context and the online setting:

*The face-to-face interactions between classmates and teachers in the classroom made me feel better involved and engaged. I could get the immediate feedback and support from my teacher and my peer students. The collaborative activities made all the members move forward hand in hand. That kind of feeling was so fabulous. However, I didn’t have a sense of belonging while learning online. We had very few cooperations and interactions with each other, and I didn’t know what my peer students were exactly doing.*

What Participant H had described seemed to indicate that the social interactions in the traditional classroom setting could enhance her enthusiasm and motivation, or to be more specific, the cooperative learning practices assisted to accelerate the motivational momentum of her DMCs in English learning. Such a result is consistent with the finding that cooperative learning can contribute to greater achievements and productivity (Laal and Ghodsi, 2012). Given that DMCs can channel learners toward a finite future target and propel them to be achievement-oriented, it is no wonder that the participants caught in DMCs could enjoy an intensified sense of achievement through the cooperative learning in conventional classroom instruction.

### Theme 3: Exam pressure

A third salient theme emerging from the analyses of interview data was exam pressure. Apart from the formal IELTS test, participants were supposed to take many other English exams or quizzes during the whole academic year. There were three English proficiency tests, including National College English Test Band 4 (CET4), National College English Test Band 6 (CET6), and National English Contest for College Students (NECCS). Two English achievement tests were College English final examinations at the end of two semesters. All the tests and exams were compulsory and the relative scores would determine the participants’ final grade-point and whether they could get the scholarship. It is thus not surprising that English exams might exert pressure on the participants’ motivational experiences. Nearly all participants reported the increasing intensity of motivation with the approaching of various English exams (see Figure 3). Participant B made the following comment:

*Our English teachers always encouraged us not to worry about CET4 or CET6 since we were preparing for the IELTS test. However, in my opinion, each exam was very important. I thought the final score was the best testimony to check out my devoted time and efforts. I got greatly motivated and devoted myself to preparing for each exam.*

Nearly all participants mentioned the contribution of various exams to their motivation growth. Participant F explained how her motivation surged with the approaching of exams:

*Generally, exams could make me anxious because I worried about doing bad. But the hard work in preparing for the IELTS test provided me the chance of doing good in many other English tests. The good scores of English exams could bring me a sense of achievement and the exam pressure thus became the magic power to push me forward.*

**Figure 4:**
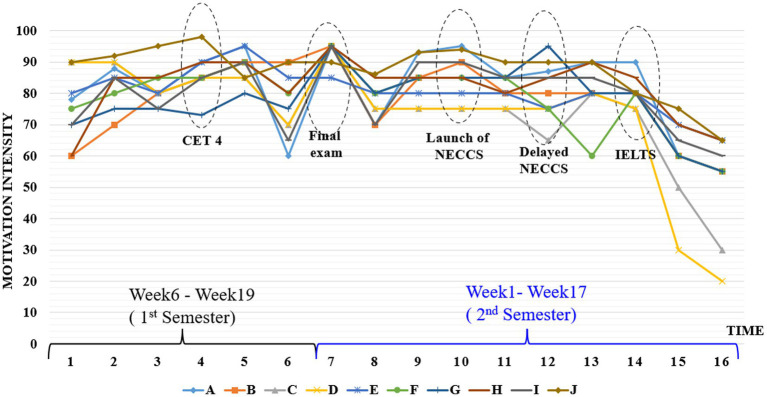
Relationship between exams and motivational surges.

The potential bearing between various English exams and the participants’ motivation growth in Figure 4 indicate that the closer the exam approached, the higher the intensity of participants’ English learning motivation would become. However, one interesting point to be noted in Figure 4 is that there was a general declining tendency in participants’ motivation trace after the ending of each exam, particularly at the end of the second semester. This means while the participants got highly motivated in dealing with the exam issues, their enthusiasm and motivation diminished considerably after they passed the relative English exams. Such findings provided further evidence for the recognition of a discernible ending point of DMCs construct.

## Discussion

The aim of the present study was 2-fold: to explore the real-time process of Chinese tertiary-level EFL learners’ motivational experience in attending the IELTS training courses and to identify the necessary parameters contributing to such long-term periods of the intense motivational experiences.

As for the first research question, it was found that 10 participants experienced the powerfully long-term motivational currents in their English learning process, and that there did exist a salient facilitative structure upholding such intense and positive emotional surges. The intensity of the participants’ English learning motivational stories, either reading English aloud every morning, working late into the night for English reading and writing, getting engaged in English study rather than indulged in playing computer games, or setting the routine schedule for English study, provides the evidence of the participants’ capacity to concentrate on studying over a sustained period of time at levels much higher than what can be normally experienced or believed possible, once they are caught up in DMCs.

The analyses of participants’ motivational experiences indicate the huge power of participants’ personal well-defined goals in English learning. Whatever it was the improvement of a particular English skill or the overall English proficiency, passing the IELTS test or landing an ideal job, such clearly-defined targets could set the orientation for the participants’ DMCs and propel them to struggle for the future vision. What needs to be pointed out is that Chinese participants with well-defined visions could get highly motivated in the traditional classroom context as well as the online learning environment. Such a finding verified the presence of salient motivational structure in participants’ hope for English language learning, supporting the claim that hope plays an important part in facilitating learners’ language learning motivation under both face-to-face and online learning environment (Marchand and Gutierrez, 2012; Yang et al., 2021). Furthermore, the participants’ motivational stories of attending the IELTS training courses indicated that they enjoyed the strong personal pleasures in accomplishing some subgoals or obtaining encouragements from others. On such a long journey, various tangible progress indicators, either in the form of the participants’ self-appraisals, the satisfying results of English exams, or by means of positive feedback from teachers, made the participants’ motivated behaviors sustained and orient their motivation flows down the way to the final vision.

All in all, the major features of 10 Chinese tertiary-level EFL learners’ motivational experiences were in accord with the key characteristics of DMCs and the overall findings showed consistency with the results previously obtained in other language learning contexts (e.g., Henry et al., 2015; Ibrahim, 2016b; Zarrinabadi and Tavakoli, 2017), yielding additional Chinese evidence for the validity of DMCs construct. The fact that 10 highly motivated participants managed to achieve their final targets further indicates that building a future self-vision and raising self-aspirations could bring benefits for language learning. Such a finding supports the assumption that the highly intense engagement in a project would eventually lead to a better learning result (Dörnyei et al., 2016).

With regard to the second research objective, the real-time data analyses of the participants’ DMCs revealed that a clear-defined target was one of the significant triggering factors for Chinese tertiary EFL learners’ motivational surges, which is congruent with the argument that a well-defined goal is a prerequisite for the initiation of DMCs (Muir and Dörnyei, 2013). Apart from the well-defined vision, some factors were identified to sustain the flow of participants’ DMCs over a long period of time. Such influencing factors consisted of different important others (e.g., peer students, teachers, and family members), social-situational factors (e.g., instructional climate) as well as exam pressure. These findings agreed with those of Zarrinabadi and Khodarahmi (2021) who found that salient others and social-situational factors (i.e., responsibility) triggered the motivational currents in learners who had experienced DMCs while being engaged in language learning. Some similar parameters, like exam pressure and classroom climate, were identified to contribute to the finding that the participants’ DMCs experiences were analogous to those described by Sak (2019).

Despite the potential pressure exams might bring about, some participants expressed their positive comments on exams in this study. To be more precise, rather than the normally expected adverse impact, some participants considered various English exams as the effective driving power for their long-term motivations. Just as participant F argued, the satisfying scores and the possible knowledge gaps she derived from various English exams boosted her perseverance in English study. The real-time data analyses shed light into the important role of participants’ self-awareness in doing something worthwhile in contributing to the DMCs birth as well as in channeling the participants’ motivated currents.

The most striking result emerging from the real-time data analyses was the dual effect of instructional contexts on the participants’ DMCs experience. The conventional instructional climate in classroom was prone to provide the driving force for participants’ motivations while the online instructional climate was likely to bring about certain impairing effects even for the participants caught up in DMCs. In this regard, this study makes an original contribution to the growing area of L2 DMCs research by suggesting a fresh comparative perspective to explore leaners’ DMCs within different learning environments. Participants’ complaints about the authoritarian teaching style, predetermined language activities, and insufficient interactions in online context, not only revealed the objective challenges of online learning in inhibiting participants’ motivation and engagement, but also highlighted teacher’s important role of building a harmonious community and designing the collaborative learning activities to promote learners’ motivated engagement. It could be suggested that a stimulating and interactive environment as well as well-designed language activities are essential for the learners’ DMCs to occur.

Despite the potential impairing impacts of the online instructional climate, most participants could still be stimulated and stay highly motivated, providing the confirmatory evidence that motivated learners were more prone to undertake challenging activities, to be actively engaged, and to exhibit enhanced performance and persistence (Schunk et al., 2008). Such a finding supports that DMCs, as a unique type of motivational engagement, is an independent and valid psychological phenomenon (Ibrahim, 2017; Muir, 2020; Zarrinabadi and Khodarahmi, 2021). Meanwhile, this finding provides further support for the contemporary view of situated motivation, which emphasizes the complexity and dynamic interplay between the environmental factors and individuals’ motivation (Brophy, 2008), and accords with the claim that DMCs are consciously initiated by something specific rather than just drifting into being (Dörnyei et al., 2014). An implication of this finding lies in its possible contribution to developing motivational theories based on the integration between individuals’ motivation to learn and the technology-mediated environments.

## Conclusion

Through the real-time data collection and analyses, the present study explored the motivational fluctuations of 10 highly motivated Chinese tertiary-level EFL learners and investigated the possible influencing factors of their emotional changes. It was found that the proposed structure of DMCs could be confirmed with Chinese tertiary-level EFL learners’ English motivation experience, providing further evidence in validating the construct of DMCs in the Chinese EFL context. As for the potential factors, important others, instructional contexts as well as the pressure of various exams were detected as the major parameters to impact Chinese tertiary-level EFL learners’ DMCs-type motivations.

The findings reported in the present study may provide both theoretical and pedagogical implications. Theoretically, the findings may empirically support DMCs as a novel construct in the framework of motivation theories and validate the contemporary “person in context” view of situated motivation. Practically, the findings might contribute to the new insights on how to leverage motivational theories to optimize the classroom language instruction as well as the online language learning. EFL teachers and instructors need promote facilitating factors of DMCs as much as possible in the instructional settings, fine-tune the language classes to eliminate possible demotivating factors, and strike a balance between these interacting factors. Considering the vital role of instructional climate in stimulating the longevity of learners’ DMCs flow, a friendly and interactive classroom atmosphere should be constructed and language courses should be designed in a way that foster learners’ senses of achievement and boost their specific L2 visions in order to trigger the birth of learners’ DMCs and inhibit the disruption in their DMCs experiences. Moreover, given that exams could assist the sustaining of learners’ motivational momentum with an increased sense of achievement, regular tests or quizzes should be administrated as an essential part of language instruction.

The current study was not without limitations. The major limitation concerns the self-reported accounts of participants. Although reflective journals and the self-assessment of the motivation intensity were conducted during the whole academic year to collect the real-time data, all the data analyses were, to a greater extent, based on the participants’ self-reported accounts, which may produce some misleading information. To yield more precise findings, the triangulation approach could be adopted by using multiple methods, like class observation and some psychological research methods. Another point to be noted is that although the findings of this study expand our understanding of the DMCs construct and provide empirical evidence about what factors contribute to learners’ DMCs experience in the Chinese EFL context, how and to what extent these triggers will affect the sustainability and strength of individual’s DMCs experience remains to be uncovered. Future research could extend the findings by exploring the variability of L2 learners’ DMCs experience through the lens of individual differences and put forward relative effective language instruction interventions as well.

## Data availability statement

The original contributions presented in the study are included in the article/Supplementary material; further inquiries can be directed to the corresponding author.

## Ethics statement

Ethical approval was not required for the study on human participants in accordance with the local legislation and institutional requirements. The participants provided their written informed consent to participate in this study.

## Author contributions

XH and DZ discussed the idea and design of the study and together revised the draft of the manuscript. XH and CW collected the data and drafted the manuscript. All authors contributed to the article and approved the submitted version.

## Funding

This study was supported by 2021 Philosophy and Social Science Fund from Jiangsu Provincial Department of Education, China (2021SJA2093) and the Scientific and Technological Innovation Team of Jiangsu University of Science and Technology (2020) for the research, authorship, and/or publication of this article.

## Conflict of interest

The authors declare that the research was conducted in the absence of any commercial or financial relationships that could be construed as a potential conflict of interest.

## Publisher’s note

All claims expressed in this article are solely those of the authors and do not necessarily represent those of their affiliated organizations, or those of the publisher, the editors and the reviewers. Any product that may be evaluated in this article, or claim that may be made by its manufacturer, is not guaranteed or endorsed by the publisher.

## Footnotes

^1^ https://www.iflyrec.com/

^2^ www.pigai.org
