# Supplementary material for: Exploring the motivational states of English learning among Chinese EFL learners at tertiary-level: A perspective of Directed Motivational Currents
Source: Front Psychol. 2022 Dec 30;13:1041258. doi: 10.3389/fpsyg.2022.1041258 (PMC9838192; doi:10.3389/fpsyg.2022.1041258)
Supplement: Supplementary file 1 [file Data_Sheet_1.docx]

**Appendix1 Outline of semi-structured interviews**

1. What is your goal for attending the IELTS training course? What are the possible reasons for you to set the target?

2. Did you have any sub-goals in preparing for the IELTS test? How did you feel when you accomplish them? Please give an example.

3. Did you have any special daily or weekly schedules to practice different language skill? If so, how did you make the arrangement? Please five some examples.

4. Who made the great influence on your determination to learning English well?

5. How did you feel when you were working hard for the IELTS test? Could you feel the progress in your English learning process?

7. Did you think the instructional atmosphere in traditional classroom differ from that in online setting? Did these differences impact your English learning motivations?

8. What were the differences of task design and language activities arrangement between the face-to-face classroom instruction and the online setting? Did these differences impact your English learning motivations?

9. Did the interaction modes in the face-to-face classroom instruction and the online setting make a difference on your English learning motivations?

10. When the English exam was approaching, did you become nervous? Do you think various exams were useful for your English learning?

11. Did you pass ILETS test successfully? Would you still devote yourself to English learning as hard as you did before? If not, why?
