# Supplementary material for: Exploring the motivational states of English learning among Chinese EFL learners at tertiary-level: A perspective of Directed Motivational Currents
Source: Front Psychol. 2022 Dec 30;13:1041258. doi: 10.3389/fpsyg.2022.1041258 (PMC9838192; doi:10.3389/fpsyg.2022.1041258)
Supplement: Supplementary file 2 [file Data_Sheet_2.docx]

Appendix 2 Examples of the core hallmarks of DMCs in participants’ motivation experience

| **Participant** | **Goal/vision** | **Salient/facilitative structure** | **Positive emotionality** |
| --- | --- | --- | --- |
| A | I wanted to find a well-paid job in a famous foreign company someday. | I knew that I was making progress and that kind of feeling was so great. And I was determined to go on reading aloud and listening more in the morning. | Whenever I think about my future job, I felt very happy and excited. These good feelings made me strong and push me forward to my target. |
| B | I want to speak fluently, listen well, read well and be good at writing. | I attended the classroom instructions in the day time. I went to the classroom for self-study in the evening. I also practiced English listening before I went to bed. I repeated these activities every day. | I felt so great to make progress every day. From the initial timidity to the present self-confidence, I was pretty sure that I was doing the right thing. All my hard work had been paid off and I was determined to go on my efforts. |
| C | Going to a foreign university and attending the international conference was my big dream. | My oral English teacher often gave positive feedback to my presentation and praised my pronunciation and intonation. That was really encouraging. | My teacher told me that I looked energetic and passionate in speaking English. I thought he was right. The confident feeling was fantastic! |
| D | I hope to improve my English writing ability. | I was so excited to see the nice comments in my writing paper. My teacher’s comments were the best encouragement to push me forward. | I could feel my progress. I enjoyed whatever I did. That kind of feeling was wonderful. |
| E | To speak English as fluently as a native speaker does is my big dream. | I got up early in the morning, practiced reading English and listened to English news. I did this practice as regularly as I can. | I enjoyed my English learning. Trying my best for my goal made me fulfilled and pleasant. |
| F | I wanted to improve my writing skills under the guidance of my English teacher. | Every morning, instead of lying in bed lazily, I got up early and practiced reading English or listening to some English newscast. | I was so excited when I found I could memorize difficult words, speak English better and understand English lecture more. I felt my progress and I believed I could learn English well. |
| G | I love English songs and English movies. I want to be able to sing a beautiful English song in public one day. | I installed English news apps on my mobile phone. Whenever I waited in line for meals or buses, I would open the news website and browse the English news. | I was not bored. I was busy in doing various assignments. It was a great experience to fight for my dream and I felt I really had a very meaningful life. |
| H | Speaking English fluently and communicating with foreigners confidently was my goal. | I devoted more time to reading aloud and imitating the native speakers' intonation and pronunciation every day. | This period was the best time in my life. It was full of positive feelings. I felt I was making progress every day. |
| I | I wanted to become an oversea university student as outstanding as those alumni. | My friends felt it unbelievable that I would concentrate on English study rather than play online games. When they found out my firmness and perseverance, they did not invite me for games any more. | My friends told me that I looked very joyful and passionate in English learning. They were right. I really enjoyed those days. |
| J | I wanted to improve my writing ability. To have a better command of authentic sentence structures and the paragraph development skills was my goal. | The scores for my English writing graded by Pigai encouraged me a lot. The better scores were the convincing signs for my progress and stimulated me a lot to practice writing harder. | I set some small goals in order to pass IELTS test. I was so happy to have my goals achieved one by one. The feeling of coming closer to your final dream was so exciting. |
